# Prognostic Value of Left-Ventricular Filling Pressure Estimated by Cardiovascular Magnetic Resonance in Patients With Acute ST-Segment Elevation Myocardial Infarction

**DOI:** 10.31083/RCM47561

**Published:** 2026-05-13

**Authors:** Weibo Li, Kairui Bo, Zhen Zhou, Yifeng Gao, Sha Li, Yue Ren, Hui Wang, Lei Xu

**Affiliations:** ^1^Department of Radiology, Beijing Anzhen Hospital, Capital Medical University, 100029 Beijing, China

**Keywords:** left-ventricular filling pressure, cardiovascular magnetic resonance, ST-segment elevation myocardial infarction, risk stratification

## Abstract

**Background:**

Left-ventricular filling pressure estimated using cardiovascular magnetic resonance (LVFP_cmr_) provides a noninvasive measure of diastolic function and has demonstrated prognostic value comparable to invasive assessment in heart failure populations. However, data on LVFP_cmr_ in patients following acute ST-segment elevation myocardial infarction (ASTEMI) are limited. Thus, this study aimed to evaluate the diagnostic and prognostic implications of LVFP_cmr_ in a cohort of patients with ASTEMI.

**Methods:**

This study included 296 patients with ASTEMI who underwent cardiovascular magnetic resonance (CMR) after percutaneous coronary intervention (PCI). The primary clinical endpoint was major adverse cardiac events (MACEs), defined as a composite of death, reinfarction, and heart failure. Univariable and multivariable Cox regression analyses were used to determine the association between LVFP_cmr_ and MACEs. Receiver operating characteristic curve and Kaplan-Meier analyses were performed to evaluate the prognostic value of LVFP_cmr_ in patients with ASTEMI.

**Results:**

During a median follow-up of 1563 days (interquartile range: 1442–1714 days), 38 patients (12.84%) experienced MACEs. These patients exhibited significantly higher CMR-derived LVFP_cmr_ values than those without MACEs (14.57 [13.17–15.99] vs. 13.30 [12.05–14.51] mmHg; *p* < 0.001). Moreover, the Youden index identified an optimal LVFP_cmr_ cutoff of 14.30 mmHg for high-risk classification (*p* < 0.001). In univariable Cox regression analysis, each 1 mmHg increase in LVFP_cmr_ was associated with a significantly higher risk of MACEs (hazard ratio [HR]: 1.31; 95% confidence interval [CI]: 1.14–1.51; *p* < 0.001). This association remained robust in multivariable models after adjustment for baseline covariates, left-ventricular ejection fraction, and infarct size (% of LV mass) (HR: 1.25 per 1 mmHg increase; 95% CI, 1.07–1.46; *p* < 0.01). The multivariable regression model yielded a Harrell C-index of 0.77, indicating strong discriminative ability for predicting MACEs.

**Conclusions:**

LVFP_cmr_ independently predicts long-term MACEs after ASTEMI, supporting the use of this approach in post-PCI risk stratification.

## 1. Introduction

Acute myocardial infarction (AMI) remains one of the leading causes of morbidity and mortality worldwide [1, 2, 3]. Following AMI, necrosis and inflammation trigger adverse ventricular remodeling, which is characterized by myocardial fibrosis, increased ventricular stiffness, and impaired diastolic relaxation [4, 5, 6]. This remodeling increases left-ventricular filling pressure (LVFP), a key marker of diastolic dysfunction, which can cause pulmonary congestion and adverse outcomes even in patients with preserved ejection fraction [7]. Elevated LVFP has also been shown to independently predict rehospitalization and major adverse cardiovascular events (MACE) in heart failure cohorts [8].

Historically, the measurement of LVFP has relied on invasive catheterization methods such as right heart catheterization (RHC), which is regarded as the gold standard for detailed hemodynamic assessment [9]. However, the invasive nature of these procedures limits their routine application in patients with AMI [10]. Therefore, noninvasive techniques, particularly transthoracic echocardiography (TTE), have become widely used for initial LVFP assessment. Nonetheless, it cannot assess myocardial tissue characteristics, which are essential for comprehending the underlying pathology in AMI [11].

Cardiovascular magnetic resonance (CMR) not only offers superior tissue characterization and functional imaging but also captures dynamic changes in myocardial structure and function, providing a more comprehensive assessment of cardiac pathology [12, 13]. CMR can quantify multiple parameters of left ventricle (LV) diastolic function, similar to echocardiography. These include strain-based myocardial deformation analysis and phase-contrast evaluation of transmitral or pulmonary venous flow. Such measurements usually require advanced image postprocessing and often involve specific pulse sequences [14]. Recently, a CMR-based estimate of LVFP (LVFP_cmr_) has been introduced, derived exclusively from routine cine images and not requiring additional sequences or scan time. This method has demonstrated considerable prognostic value in patients with heart failure, potentially offering an accurate and noninvasive alternative for LVFP assessment [15]. Given its accessibility from standard cine CMR sequences, LVFP_cmr_ may be feasibly incorporated into routine post–percutaneous coronary intervention (PCI) imaging protocols to facilitate early identification of high-risk patients, optimize follow-up frequency, and guide adjunctive therapeutic decisions. Nevertheless, the clinical utility and prognostic significance of LVFP_cmr_ in acute ST-segment elevation myocardial infarction (ASTEMI) are yet to be elucidated. Considering its potential advantages, this study aimed to assess the prognostic value of LVFP_cmr_ in patients with ASTEMI who had undergone PCI.

## 2. Materials and Methods

### 2.1 Study Population

Consecutive patients admitted to the coronary care unit of Anzhen Hospital with an initial diagnosis of ASTEMI were screened for inclusion. The study was conducted in accordance with the Declaration of Helsinki and approved by the Ethics Committee of Beijing Anzhen Hospital. Written informed consent was obtained from all participants. Between June 2016 and December 2022, a total of 296 patients who underwent gadolinium-enhanced CMR following primary PCI were evaluated. Inclusion criteria: (1) diagnosis of ASTEMI according to the Fourth Universal Definition of Myocardial Infarction [16] and (2) completion of CMR within 3–7 days of hospital admission. Exclusion criteria: (1) known cardiomyopathy, congenital or valvular heart disease, pericardial disease, or severe arrhythmia; (2) incomplete or missing essential laboratory data including brain natriuretic peptide (BNP) and creatine kinase-myocardial band (CK-MB); (3) insufficient follow-up information; and (4) poor image quality.

### 2.2 CMR Protocol

All examinations were performed on 3.0 T cardiovascular MR systems, including the Achieva (Philips Healthcare, Best, Netherlands) and the Discovery MR750w (GE Healthcare, Milwaukee, WI, USA), both equipped with a 32-channel phased-array cardiac coil, electrocardiographic (ECG) gating, and respiratory navigation. The standard imaging protocol featured steady-state free-precession (SSFP) cine sequences during breath-holding, T2-weighted short-axis imaging, and late gadolinium enhancement (LGE). A cine CMR study was conducted using an SSFP sequence, which captured contiguous short-axis images from the mitral annulus to the apex, covering both ventricles. Long-axis views in two-, three-, and four-chamber planes were also acquired, with 25–30 reconstructed cardiac phases per cycle. T2-weighted short-axis imaging used a short tau inversion recovery technique. Myocardial perfusion data were collected during the intravenous administration of 0.1 mmol/kg gadolinium-based contrast at a rate of 4 mL/s. For LGE, prospectively ECG-gated gradient-echo images in short- and long-axis planes were obtained 10–15 min after injecting 0.2 mmol/kg of contrast (TR/TE = 4.1/1.6 ms; flip angle = 20°; matrix size = 256 × 130).

### 2.3 CMR Analysis

All CMR datasets were transferred to a dedicated workstation and analyzed using commercial software CVI42 (version 5.2.0, Circle Cardiovascular Imaging Inc., Calgary, Canada). Ventricular function was evaluated using the Short 3D module, which semiautomatically traced endocardial and epicardial borders at end-diastolic and end-systolic frames on cine short-axis stacks, including papillary muscles. Contours were visually inspected and manually adjusted by two experienced cardiovascular radiologists (>10 years of experience). Quantitative indexes, including left-ventricular ejection fraction (LVEF), end-diastolic volume (LVEDV), end-systolic volume (LVESV), stroke volume (LVSV), and left-ventricular mass (LVM), were automatically derived by the software. Left-atrial volume (LAV) was obtained by manual endocardial contouring in both four- and two-chamber views, and the maximal LAV was calculated at LV end-systole (just before mitral valve opening) using the biplane area-length method.

LGE was identified as regions with signal intensity of >5 standard deviations above normal myocardium on short-axis images. The infarct size was expressed as LGE mass normalized to the total LV mass. Hypointense cores within areas of hyperenhancement on LGE, indicating microvascular obstruction (MVO), were classified and reported as a percentage of LV mass. Zones of hypointensity on T2-weighted short tau inversion recovery sequences that match infarct regions were considered to represent intramyocardial hemorrhage (IMH).

### 2.4 Estimating Pulmonary Capillary Wedge Pressure From CMR

A CMR-based model estimating LVFP from LAV and LVM has been recently proposed. The equation was developed using data from a cohort of 835 individuals evaluated for suspected heart failure who underwent CMR, echocardiography, and invasive hemodynamic assessment [15]. In this study, the formula was applied to calculate LVFP values using measured LAV and LVM:

LVFP_cmr_ = 6.1352 + (0.07204 × LAV) + (0.02256 × LVM)

### 2.5 Clinical Endpoints and Outcome

Composite endpoint events were identified by reviewing Anzhen Hospital’s electronic medical records, with additional follow-up via telephone for events that occurred after discharge. MACE was defined as a composite endpoint including all-cause death, recurrent myocardial infarction, or heart failure requiring rehospitalization following AMI. If a patient experienced multiple events, only the most severe one was recorded, based on the following hierarchy: death, reinfarction, then heart failure. Each patient was counted once for a MACE event in the analysis.

### 2.6 Statistical Analyses

Continuous data were expressed as the mean ± standard deviation when normally distributed and as the median with interquartile range (IQR) when non-normally distributed. Categorical variables were presented as counts and percentages. Baseline characteristics between patients with and without MACE were compared using the independent *t*-test or Wilcoxon rank-sum test for continuous variables and the chi-square test for categorical variables. Associations between continuous parameters were evaluated using Spearman’s rank correlation. The discriminative performance of LVFP_cmr_ in predicting MACE was determined by performing receiver operating characteristic (ROC) analysis, where the area under the curve (AUC) and Youden index were used to calculate the optimal cutoff. Event-free survival was analyzed using the Kaplan-Meier method, and intergroup differences were assessed with the log-rank test. Univariate Cox proportional hazards models were used to estimate hazard ratios (HRs) for MACE and mortality. Variables with *p* < 0.05 in univariate testing were subsequently entered into multivariable Cox regression using a stepwise selection approach, and those with *p* < 0.05 were retained. In addition, a hierarchical composite outcome analysis was performed using the win ratio (WR) method (death > reinfarction > heart failure hospitalization). Patients were compared in all possible unmatched pairs. A pair was declared a “win” for the patient when a higher-priority event occurred later, and ties moved on to the next level. The WR was calculated as total wins divided by total losses, with 95% confidence intervals (CIs). All statistical analyses were performed using SPSS (version 29, Statistical Package for the Social Sciences, International Business Machines, Armonk, NY, USA) and R software (version 4.4.2; R Foundation for Statistical Computing, Vienna, Austria).

### 2.7 Sex-Specific CMR-Derived Pulmonary Capillary Wedge Pressure (PCWP) Sub-Analysis

A sex-specific CMR-derived PCWP (sex-specific LVFP_cmr_) was additionally computed from LAV and LVM, with sex coded as female = 0 and male = 1, as proposed by Garg *et al*. [17]: Sex-specific LVFP_cmr_ = 5.7591 + 0.07505 × LAV + 0.05289 × LVM – 1.9927 × sex. The equation was validated using PCWP measurements obtained via RHC in heart failure cohorts. Compared with the generic equation, it demonstrated a better reduction of sex-related bias and enhanced the prognostic accuracy [17]. Using identical censoring rules, endpoints, and covariate adjustment as the main analysis, univariable and multivariable Cox models were fit, replacing the generic LVFP_cmr_ with the sex-specific LVFP_cmr_. Subsequently, the optimal cut-point was derived via the Youden index for Kaplan-Meier curves. To avoid multicollinearity, entering the generic and sex-specific metrics in the same model was avoided.

### 2.8 Summary of Study Design and Analytical Approach

In summary, this single-center retrospective study included 296 patients with ASTEMI who underwent CMR after primary PCI. Quantitative CMR parameters such as LAV, LVM, and derived LVFP_cmr_ were examined in relation to future MACE. The prognostic value of LVFP_cmr_ was evaluated using Kaplan-Meier survival curves and Cox regression, adjusting for key clinical and imaging factors. These analyses aimed to determine if LVFP_cmr_ provides independent prognostic insights beyond conventional measures, such as LVEF and infarct size.

## 3. Results

### 3.1 Patient Characteristics

**Fig. 1.**
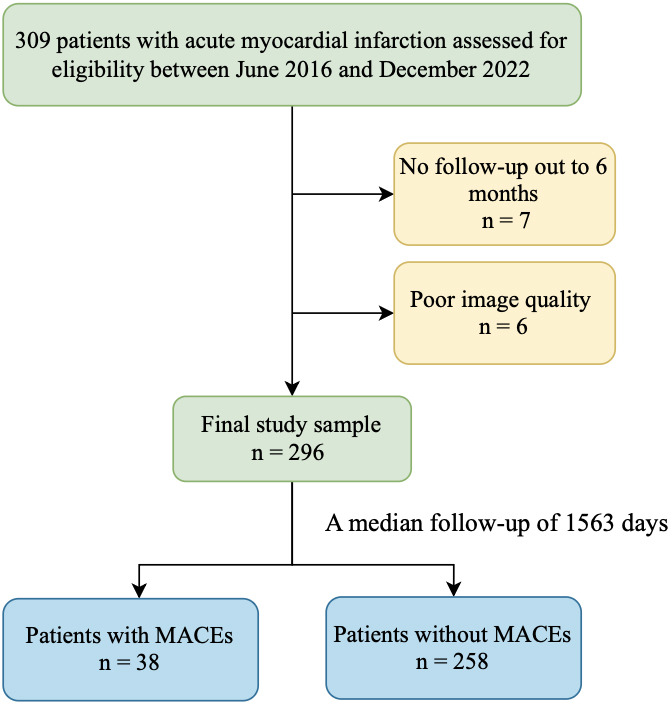
**Flowchart of patient inclusion**. MACE, major adverse cardiovascular events.

A total of 309 patients initially met the inclusion criteria (Fig. 1). Of these, 7 lacked 6-month follow-up data, and 6 had poor CMR image quality. The final cohort comprised 296 patients, with a median age of 58 years (IQR, 49–66); 248 (83.78%) were male, and 48 (16.22%) were female. The last follow-up was in December 2024, with a median duration of 1563 days (IQR, 1442–1714). Of these, 38 patients (12.84%) experienced MACE, including 8 deaths from all causes, 12 reinfarctions, and 18 hospitalizations for heart failure. The primary baseline clinical and CMR characteristics are summarized in Tables 1,2.

**Table 1.** **Baseline characteristics of the study population**.

Parameter			All participants (n = 296)	No MACE (n = 258)	MACE (n = 38)	*p* value
Baseline characteristics						
	Age (y)		58.00 (49.00, 66.00)	57.00 (48.00, 65.00)	62.50 (51.00, 66.00)	0.398
	Men		248 (83.78)	213 (82.56)	35 (92.11)	0.136
	Body mass index (kg/m^2^)		25.66 (23.55, 27.68)	25.71 (23.53, 27.68)	25.25 (23.67, 28.58)	0.494
	Heart rate (beats/min)		78.00 (70.00, 88.00)	78.00 (70.00, 87.00)	80.00 (72.00, 90.00)	0.098
	Systolic blood pressure (mmHg)		122.27 ± 17.70	122.88 ± 17.29	118.13 ± 20.03	0.172
	Diastolic blood pressure (mmHg)		77.00 (70.00, 84.00)	77.50 (70.00, 84.00)	77.00 (68.00, 84.00)	0.536
Cardiovascular risk factors						
	Previous/current smoker (%)		188 (63.51)	158 (61.24)	30 (78.95)	**0.034**
	Hypertension (%)		184 (62.16)	156 (60.47)	28 (73.68)	0.117
	Diabetes (%)		106 (35.81)	90 (34.88)	16 (42.11)	0.386
	Dyslipidemia (%)		193 (65.20)	168 (65.12)	25 (65.79)	0.935
	Prior myocardial infarction (%)		11 (3.72)	10 (3.88)	1 (2.63)	>0.99
	Previous PCI (%)		16 (5.41)	13 (5.04)	3 (7.89)	0.442
Killip class						**0.013**
	I		214 (72.30)	194 (75.19)	20 (52.63)	
	II		73 (24.66)	58 (22.48)	15 (39.47)	
	III		3 (1.01)	2 (0.78)	1 (2.63)	
	IV		6 (2.03)	4 (1.55)	2 (5.26)	
TIMI flow before PCI						0.203
	0		209 (70.61)	181 (70.16)	28 (73.68)	
	1		19 (6.42)	19 (7.36)	0 (0)	
	2		25 (8.45)	23 (8.91)	2 (5.26)	
	3		43 (14.53)	35 (13.57)	8 (21.05)	
TIMI flow grade after PCI						0.657
	2		12 (4.05)	10 (3.88)	2 (5.26)	
	3		284 (95.95)	248 (96.12)	36 (94.74)	
Location anterior (%)			138 (46.62)	114 (44.19)	24 (63.16)	**0.029**
Blood results						
	CK-MB mass (ng/mL)		218.85 (106.85, 303.00)	212.15 (105.90, 303.00)	286.60 (186.50, 303.00)	0.077
	Myoglobin (ug/L)		273.50 (76.00, 513.40)	277.50 (80.00, 503.00)	257.50 (56.10, 642.68)	0.978
	BNP (pg/mL)		177.50 (82.00, 319.50)	166.50 (81.00, 294.00)	283.00 (117.00, 486.00)	**0.004**
	Creatinine (µmol/L)		71.70 (64.00, 82.00)	71.45 (62.90, 82.00)	72.95 (64.90, 82.90)	0.678
	eGFR (mL/min/1.73 m^2^)		98.11 (88.75, 108.04)	98.77 (88.78, 108.14)	96.16 (87.66, 105.37)	0.511
	Triglycerides (mmol/L)		1.46 (1.08, 1.97)	1.48 (1.09, 2.00)	1.32 (0.85, 1.90)	0.315
	Total cholesterol (mmol/L)		4.65 (4.03, 5.47)	4.68 (4.07, 5.54)	4.31 (3.63, 5.22)	0.062
	HDL cholesterol (mmol/L)		1.04 (0.89, 1.21)	1.04 (0.89, 1.20)	1.00 (0.80, 1.26)	0.266
	LDL cholesterol (mmol/L)		3.09 (2.40, 3.67)	3.09 (2.41, 3.72)	3.09 (2.37, 3.62)	0.789
	High-sensitivity CRP (mg/L)		5.13 (2.26, 10.92)	4.58 (2.11, 10.28)	7.31 (3.45, 20.00)	**0.010**
	Blood glucose on admission (mmol/L)		8.69 (7.17, 11.90)	8.71 (7.17, 12.04)	8.66 (7.21, 10.13)	0.830
	Fasting blood glucose (mmol/L)		6.57 (5.63, 8.88)	6.48 (5.57, 8.86)	6.68 (5.89, 9.03)	0.385
	HbA1c (%)		6.00 (5.60, 7.20)	6.00 (5.60, 7.10)	6.10 (5.70, 7.30)	0.449
GRACE score			119.00 (101.00, 131.00)	116.50 (99.00, 131.00)	129.50 (114.00, 148.00)	**0.002**
GRACE risk category						**0.002**
	Low		103 (34.80)	96 (37.21)	7 (18.42)	
	Intermediate		145 (48.99)	127 (49.22)	18 (47.37)	
	High		48 (16.22)	35 (13.57)	13 (34.21)	
Door-to-wire time (min)			100.00 (88.00, 130.00)	100.00 (87.00, 132.00)	100.00 (91.00, 123.00)	0.583
Procedures						
	Number of diseased arteries (%)					0.155
		1	120 (40.54)	106 (41.09)	14 (36.84)	
		2	87 (29.39)	71 (27.52)	16 (42.11)	
		3	89 (30.07)	81 (31.39)	8 (21.05)	
	Location of culprit lesion (%)					**0.003**
		LAD	167 (56.42)	141 (54.65)	26 (68.42)	
		LCX	28 (9.46)	21 (8.14)	7 (18.42)	
		RCA	101 (34.12)	96 (37.21)	5 (13.16)	
Medication						
	Aspirin		284 (95.95)	250 (96.90)	34 (89.47)	0.054
	Clopidogrel/Prasugrel/Ticagrelor		291 (98.31)	255 (98.84)	36 (94.74)	0.125
	Statin		275 (92.91)	240 (93.02)	35 (92.11)	0.740
	ACE inhibitor/AT1 receptor blocker		171 (57.77)	144 (55.81)	27 (71.05)	0.076
	Beta-blocker		210 (70.95)	179 (69.38)	31 (81.58)	0.122
	Diuretic		52 (17.57)	44 (17.05)	8 (21.05)	0.545

Continuous variables are expressed as median (interquartile range), and categorical variables as n/N (%). Statistical significance was defined as *p* < 0.05 (bold values). BMI, body mass index; PCI, percutaneous coronary intervention; HbA1c, glycated hemoglobin; CK-MB, creatine kinase-myocardial band; BNP, brain natriuretic peptide; eGFR, estimated glomerular filtration rate; HDL, high-density lipoprotein; LDL, low-density lipoprotein; CRP, C-reactive protein; LAD, left anterior descending artery; LCX, left circumflex artery; RCA, right coronary artery; TIMI, thrombolysis in myocardial infarction; ACEI, angiotensin-converting enzyme inhibitor; ARB, angiotensin II receptor blocker. Data are presented as n/N (%) or median (interquartile range).

**Table 2.** **CMR characteristics of the study population**.

Parameter	All participants (n = 296)	No MACE (n = 258)	MACE (n = 38)	*p* value
LAV_min_ (mL)	33.16 (26.23, 43.36)	32.43 (24.96, 41.67)	41.24 (32.64, 55.22)	< **0.001**
LAV_max_ (mL)	60.21 (46.51, 75.30)	58.16 (45.66, 73.35)	71.61 (51.55, 81.65)	**0.008**
LAEF (%)	50.37 (44.60, 55.30)	50.61 (45.39, 55.97)	46.68 (38.42, 51.75)	**0.004**
LVEF (%)	48.06 ± 12.53	49.47 ± 11.53	38.49 ± 14.85	< **0.001**
LVEDV (mL)	126.38 (100.65, 152.09)	123.00 (97.35, 145.64)	148.73 (121.72, 174.10)	< **0.001**
LVESV (mL)	64.65 (45.83, 85.77)	62.70 (44.28, 80.77)	97.71 (59.12, 121.94)	< **0.001**
LVFP_cmr_ (mmHg)	13.37 (12.16, 14.72)	13.30 (12.05, 14.51)	14.57 (13.17, 15.99)	< **0.001**
Sex-specific LVFP_cmr_ (mmHg)	15.04 (12.55, 16.99)	14.78 (12.45, 16.75)	16.46 (14.69, 19.45)	< **0.001**
SV (mL)	58.00 (46.18, 70.67)	58.30 (47.00, 71.00)	55.05 (42.10, 68.40)	0.107
CO (L/min)	4.16 (3.40, 5.10)	4.18 (3.43, 5.12)	3.87 (3.29, 5.00)	0.337
LV MASS (g)	132.25 (108.50, 153.47)	129.83 (105.00, 151.34)	147.48 (123.02, 189.14)	< **0.001**
Infarct size (% LV mass)	29.72 (19.76, 37.74)	27.68 (18.48, 36.58)	35.73 (27.56, 48.04)	< **0.001**
Extent of MVO (% LV mass)	1.28 (0.00, 3.81)	1.09 (0.00, 3.54)	2.79 (0.50, 8.50)	**0.004**
IMH present (%)	165 (55.74)	138 (53.49)	27 (71.05)	**0.042**
MVO present (%)	191 (64.53)	160 (62.02)	31 (81.58)	**0.019**

Continuous variables are expressed as median (interquartile range), and categorical variables as n/N (%). Statistical significance was defined as *p* < 0.05 (bold values). CMR, cardiac magnetic resonance; LA, left atrium; LAV_min_, minimum LA volume; LAV_max_, maximum LA volume; LAEF, left-atrial ejection fraction; LV, left-ventricular; LVEF, left-ventricular ejection fraction; LVEDV, left-ventricular end-diastolic volume; LVESV, left-ventricular end-systolic volume; LVFP_cmr_, CMR-derived left-ventricular filling pressure; SV, stroke volume; CO, cardiac output; MVO, microvascular obstruction; IMH, intramyocardial hemorrhage.

### 3.2 Clinical and Cardiac MRI Characteristics Based on the Presence of MACE

Compared with the group that did not experience MACE, those with MACE displayed significantly reduced LVEF (38.49% ± 14.85 vs. 49.47% ± 11.53; *p* < 0.001). Moreover, these patients exhibited higher LVEDV (148.73 mL [121.72, 174.10] vs. 123.00 mL [97.35, 145.64]; *p* < 0.001), LVFP_cmr_ (14.57 mmHg [13.17, 15.99] vs. 13.30 mmHg [12.05, 14.51]; *p* < 0.001), and infarct size (% LV mass) (35.73% [27.56, 48.04] vs. 27.68% [18.48, 36.58]; *p* < 0.001). In addition, B-type natriuretic peptide (BNP) levels were substantially higher in the MACE cohort (283.00 pg/mL [117.00, 486.00] vs. 166.50 pg/mL [81.00, 294.00]; *p* < 0.01).

### 3.3 Correlation Between LVFP_cmr_ and CMR-Derived LA/LV Indexes

**Fig. 2.**
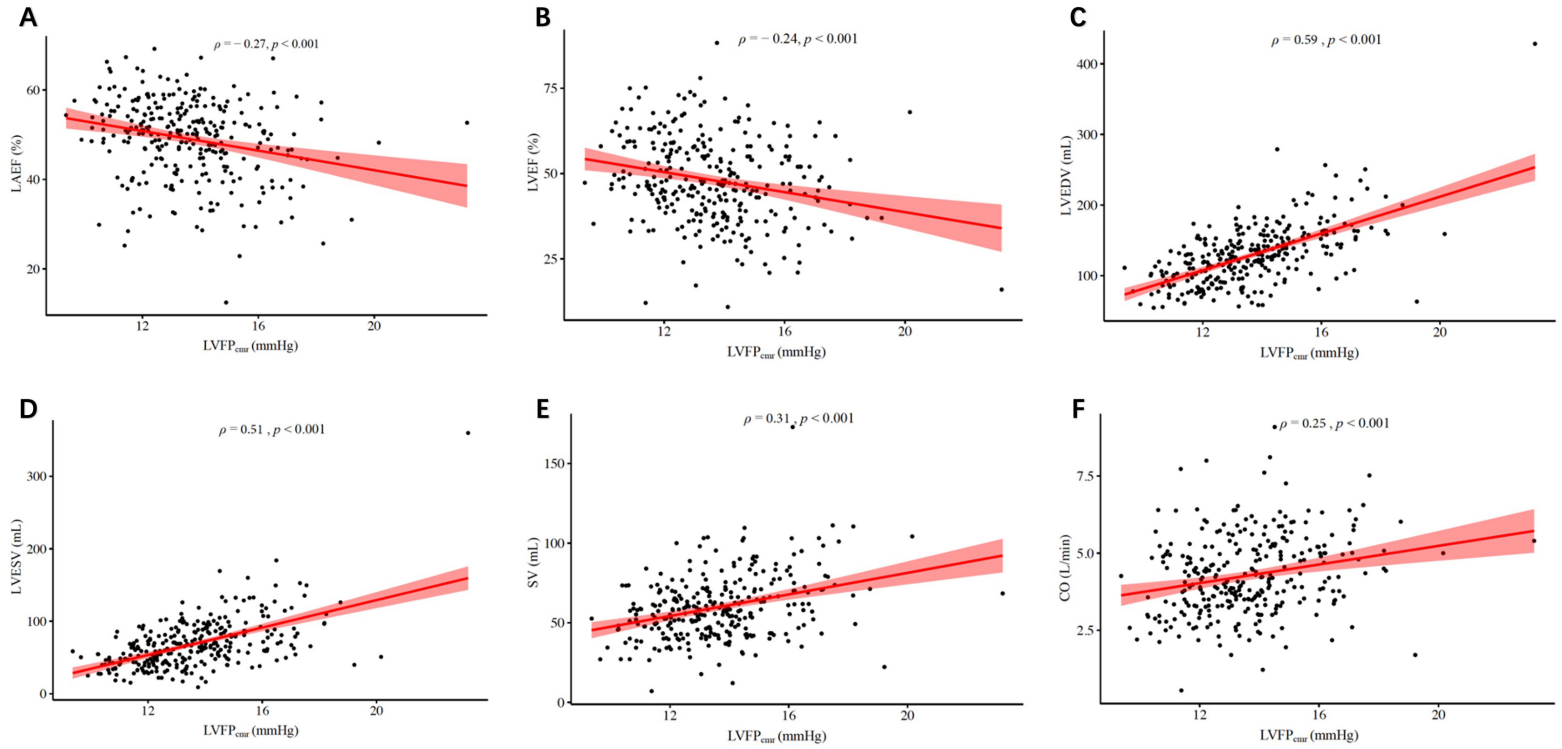
**Scatter plots showing the correlations between LVFP_𝐜𝐦𝐫_ and cardiac structural/functional parameters**. (A) LAEF, (B) LVEF, (C) LVEDV, (D) LVESV, (E) SV, and (F) CO. Spearman’s correlation coefficients (ρ) and *p* values are displayed. Shaded areas represent 95% confidence intervals of the fitted regression lines.

**Fig. 3.**
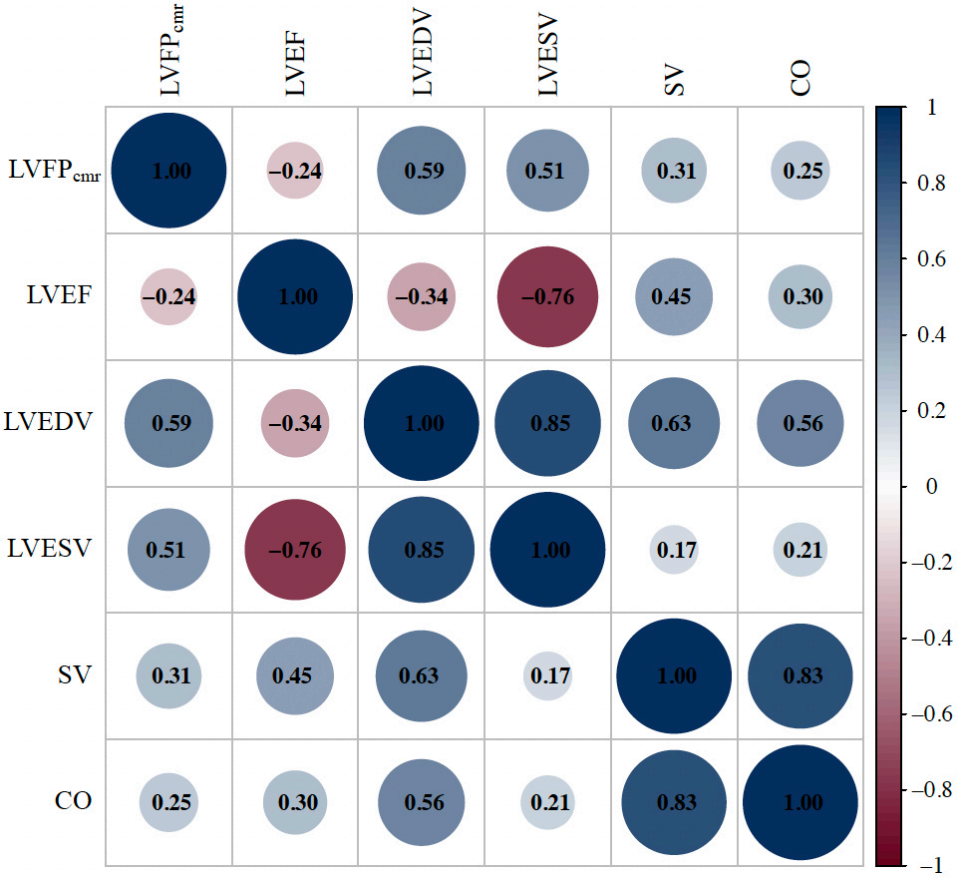
**Correlation matrix plot of LVFPcmr and key CMR-derived parameters**. The correlation matrix displays pairwise Spearman correlation coefficients among LVFP_cmr_, LVEF, LVEDV, LVESV, SV, and CO. Circle size and color intensity represent the magnitude and direction of the correlation (blue = positive; red = negative).

The cross-sectional associations between LVFP_cmr_ and chamber structure/function were explored using Spearman’s rank correlation (ρ) (Table 3). LVFP_cmr_ demonstrated the strongest correlations with LV mass (ρ = 0.62; *p* < 0.001) and LVEDV(ρ = 0.59; *p* < 0.001), followed by LVESV (ρ = 0.51; *p* < 0.001). Moderate positive relationships were noted with stroke volume and cardiac output (ρ= 0.31 and 0.25, respectively; both *p* < 0.001). As expected for a filling-pressure marker, LVFP_cmr_ was inversely associated with LAEF and LVEF (ρ = –0.27 and –0.24; both *p* < 0.001). Correlations with infarct size were small but significant (ρ = 0.15; *p* = 0.008). Measures of microvascular injury showed weak yet significant associations: extent of MVO (ρ = 0.19; *p* < 0.001), MVO present (ρ = 0.14; *p* = 0.020), and IMH present (ρ = 0.15; *p* = 0.012). Scatter plots depicting the association between LVFP_cmr_ and LA/LV structural indexes are presented in Fig. 2. Consistent with the Spearman correlation coefficients, the visual patterns suggested small to moderate monotonic relationships for most parameters. A correlation matrix plot of LVFP_cmr_ and major CMR-derived volumetric and functional parameters is shown in Fig. 3.

**Table 3.** **Associations between LA/LV structure and function derived from CMR among patients with STEMI**.

Characteristics	LVFP_cmr_	
	*ρ*	*p* value
LAEF (%)	–0.27	< **0.001**
LVEF (%)	–0.24	< **0.001**
LVEDV (mL)	0.59	< **0.001**
LVESV (mL)	0.51	< **0.001**
SV (mL)	0.31	< **0.001**
CO (L/min)	0.25	< **0.001**
LV MASS (g)	0.62	< **0.001**
Infarct size (% LV mass)	0.15	**0.008**
Extent of MVO (% LV mass)	0.19	< **0.001**
IMH present (%)	0.15	**0.012**
MVO present (%)	0.14	**0.020**

Statistical significance was defined as *p* < 0.05 (bold values). STEMI, ST-elevation myocardial infarction.

### 3.4 Association of LVFP_cmr_ With Outcomes

**Fig. 4.**
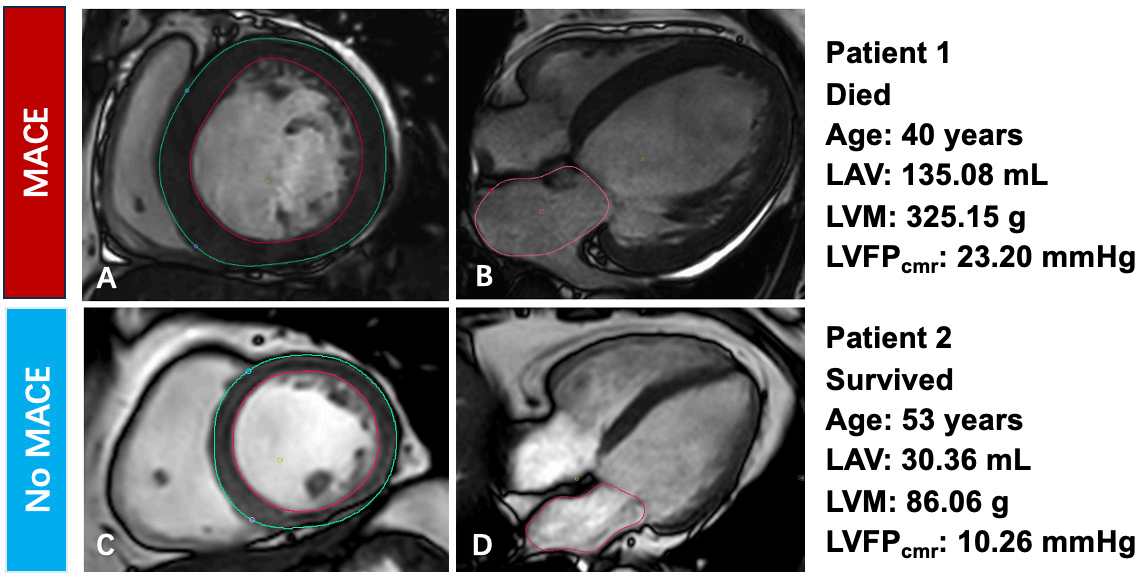
**Representative CMR findings and their relationship with MACE**. (A,B) Patient 1, who died during follow-up (age 40 years). (A) Short-axis end-diastolic cine image with LV endocardial (red) and epicardial (green) contours used to calculate LV mass (LVM 325.15 g). (B) Four-chamber cine image with left-atrial contour (pink) used to calculate left-atrial volume (LAV 135.08 mL). The derived LVFP_cmr_ was 23.20 mmHg. (C,D) Patient 2, who survived (age 53 years). (C) Short-axis cine image with LV mass measurement (LVM 86.06 g). (D) Four-chamber cine image with left-atrial contour for volume assessment (LAV 30.36 mL). The derived LVFP_cmr_ was 10.26 mmHg. LVM, left-ventricular mass.

**Fig. 5.**
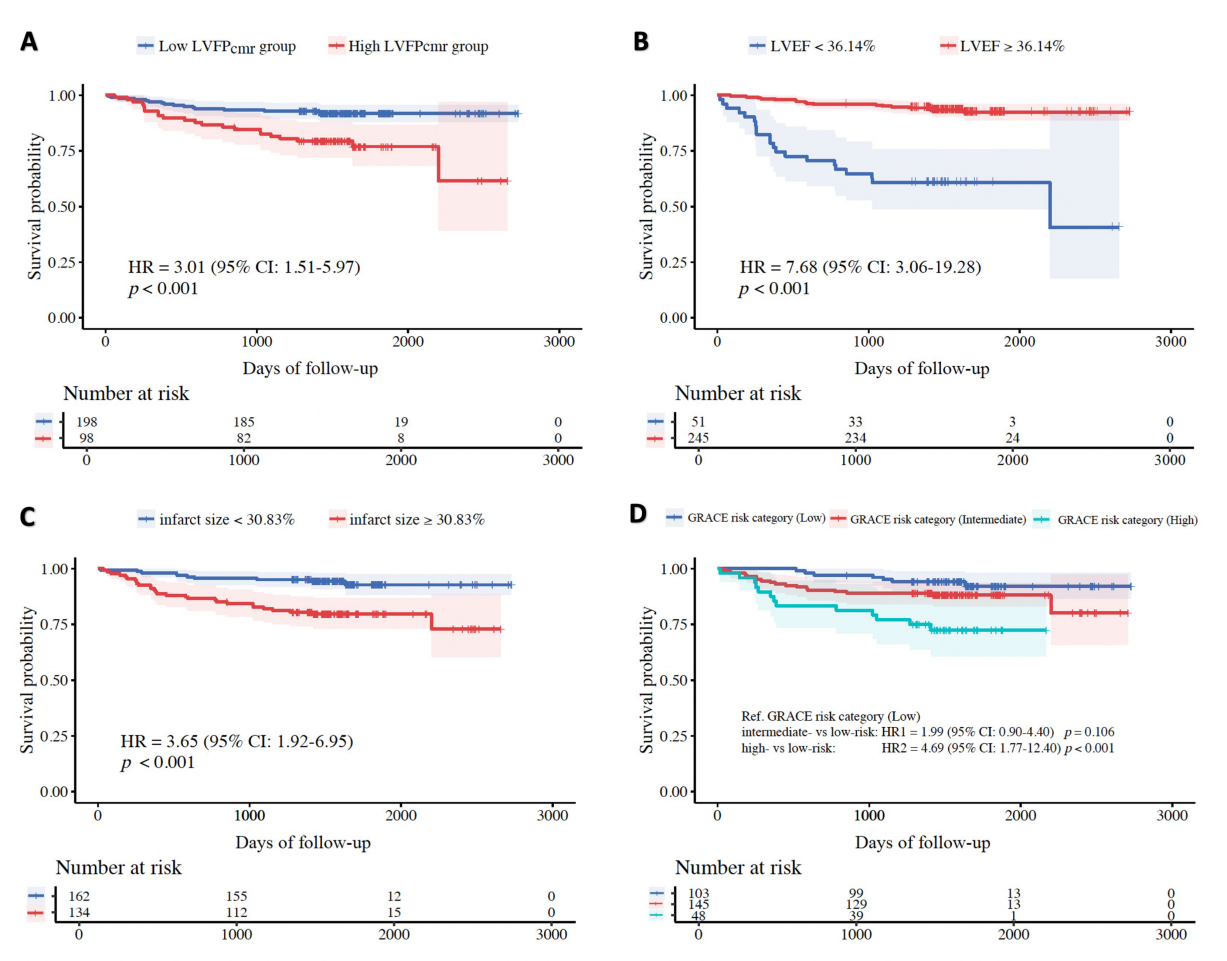
**Kaplan-Meier curves of MACE-free survival**. (A) Stratified by LVFP_cmr_ (low vs. high using the study cutoff of 14.30 mmHg). (B) Stratified by LVEF (<36.14% vs. ≥36.14%). (C) Stratified by infarct size (<30.83% vs. ≥30.83% of LV mass). (D) Stratified by GRACE risk category (low, intermediate, high). Shaded bands denote 95% confidence intervals. GRACE, Global Registry of Acute Coronary Events.

The LVFP_cmr_ demonstrated considerable variability within the cohort; the median LVFP_cmr_ value in the overall cohort was 13.37 mmHg (IQR, 12.16–14.72 mmHg). The AUC of LVFP_cmr_ for MACE was 0.67 (95% CI, 0.58–0.76). Based on ROC curve analysis, an optimal cutoff of 14.30 mmHg (sensitivity 0.58, specificity 0.71) was identified to stratify patients into a low-LVFP_cmr_ group (n = 198) and a high-LVFP_cmr_ group (n = 98). The high-LVFP_cmr_group exhibited a significantly higher incidence of MACE than the low-LVFP_cmr_ group (22.45% vs. 8.08%, *p* < 0.001). Fig. 4 presents the CMR findings and corresponding clinical data from patients with and without MACE. Kaplan-Meier curves showed significantly lower MACE-free survival in patients with higher LVFP_cmr_, lower LVEF, and larger infarct size (all log-rank *p* < 0.001; Fig. 5). Optimal cutoffs for variable dichotomization—specifically for LVEF and infarct size (% of LV mass)—were derived from ROC analysis by maximizing the Youden index in the present cohort, followed by application to Kaplan-Meier and Cox analyses.

### 3.5 Predictive Value of LVFP_cmr_

In univariable Cox regression, higher LVFP_cmr_ was significantly linked to MACE (HR, 1.31; 95% CI, 1.14–1.51; *p* < 0.001). In multivariable analysis, LVFP_cmr_ remained an independent predictor of MACE (HR, 1.25; 95% CI, 1.07–1.46; *p* < 0.01). Furthermore, LVEF (per 1% increase: HR, 0.96; 95% CI, 0.93–0.99; *p* = 0.017), infarct size (% LV mass; per 1% increase: HR, 1.03; 95% CI, 1.01–1.05; *p* = 0.017), and GRACE risk category (high vs. low risk: HR, 4.72; 95% CI, 1.77–12.19; *p* < 0.01) were independently associated with MACE (Table 4). The Harrell C-index of the model incorporating LVFP_cmr_, along with clinical and imaging covariates, was 0.77 (95% CI, 0.68–0.88), indicating robust discriminatory ability for predicting MACE.

**Table 4.** **Univariate and multivariate Cox regression analysis for MACE prediction**.

Characteristics		Univariate analysis			Multivariate analysis		
		HR	95% CI	*p* value	HR	95% CI	*p* value
BNP (pg/mL)		1.002	1.001–1.003	< **0.001**			
High-sensitivity CRP (mg/L)		1.04	1.01–1.07	**0.003**			
Previous/current smoker (%)		2.23	1.02–4.86	**0.044**			
GRACE risk category							
	Low	Ref			Ref		
	Intermediate	1.99	0.83–4.76	0.123	2.15	0.75–4.64	0.179
	High	4.86	1.93–12.26	< **0.001**	4.72	1.77–12.19	**0.001**
Killip class							
	I	Ref					
	II	2.43	1.24–4.78	**0.010**			
	III	3.77	0.50–28.16	0.196			
	IV	4.91	1.14–21.11	**0.032**			
Location of culprit lesion							
	LAD	Ref					
	LCX	1.75	0.76–4.04	0.192			
	RCA	0.31	0.12–0.81	**0.017**			
Location anterior (%)		1.96	1.01–3.82	**0.047**			
IMH (%)		2.06	1.02–4.16	**0.043**			
Infarct size (% LV mass)		1.05	1.03–1.08	< **0.001**	1.03	1.01–1.05	**0.017**
MVO (% LV mass)		1.05	1.01–1.09	**0.006**			
LVFP_cmr_ (mmHg)		1.31	1.14–1.51	< **0.001**	1.25	1.07–1.46	**0.005**
Sex-specific LVFP_cmr_ (mmHg)		1.18	1.09–1.29	< **0.001**			
LAEF (%)		0.95	0.92–0.98	**0.001**			
LVEF (%)		0.93	0.90–0.95	< **0.001**	0.96	0.93–0.99	**0.017**

Statistical significance was defined as *p* < 0.05 (bold values). HR, hazard ratio; CI, confidence interval.

### 3.6 Hierarchical Composite WR Analysis Comparing High vs. Low-LVFP_cmr_ Groups

In the hierarchical composite analysis, the high-LVFP_cmr_ group displayed significantly worse outcomes than the low-LVFP_cmr_ group. The WR was 0.35 (95% CI, 0.33–0.37), signifying that patients with elevated LVFP_cmr_ experienced more severe adverse events earlier and more frequently. These results highlight the prognostic significance of LVFP_cmr_ beyond time-to-first-event analysis.

### 3.7 Sex-Specific Sub-Analysis

**Fig. 6.**
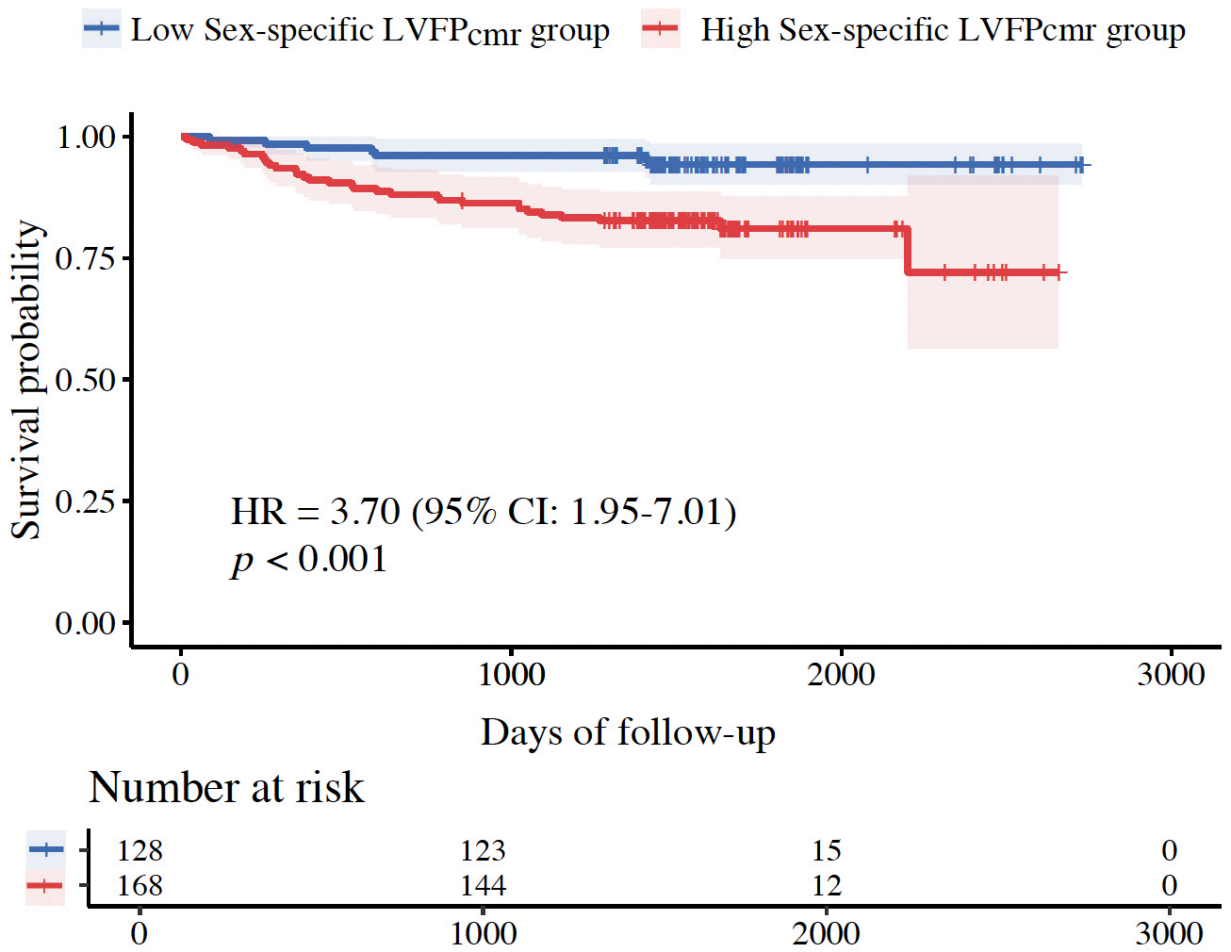
**Kaplan-Meier survival curves stratified by sex-specific LVFP_cmr_**.

The sex-specific LVFP_cmr_ equation showed that higher sex-specific LVFP_cmr_ was associated with increased risk of MACE in univariable analysis (HR, 1.18; 95% CI, 1.09–1.29; *p* < 0.001) (Table 4). In multivariable analysis, sex-specific LVFP_cmr_ remained an independent predictor of MACE (HR, 1.17; 95% CI, 1.07–1.28; *p* < 0.001) (Table 5). The AUC of sex-specific LVFP_cmr_ for MACE was 0.67 (95% CI, 0.57–0.76). The optimal cut-point (14.45 mmHg) separated Kaplan-Meier curves with significantly lower event-free survival in the high sex-specific LVFP_cmr_ group (log-rank *p* < 0.001) (Fig. 6). Therefore, the sex-specific equation yields comparable prognostic power in our ASTEMI cohort.

**Table 5.** **Multivariable Cox regression analysis using Sex-specific LVFP_𝐜𝐦𝐫_ for predicting MACE**.

Characteristics		Multivariate analysis		
		HR	95% CI	*p* value
GRACE risk category				
	Low	Ref		
	Intermediate	2.27	0.92–5.58	0.076
	High	5.43	2.07–14.25	< **0.001**
Location of culprit lesion				
	LAD	Ref		
	LCX	2.84	1.15–7.00	**0.024**
	RCA	0.55	0.20–1.54	0.256
Infarct size (% LV mass)		1.03	1.00–1.05	**0.020**
Sex-specific LVFP_cmr_ (mmHg)		1.17	1.07–1.28	< **0.001**
LVEF (%)		0.96	0.93–0.99	**0.005**

Statistical significance was defined as *p* < 0.05 (bold values).

## 4. Discussion

This study on patients undergoing CMR after ASTEMI and PCI demonstrated that the novel CMR-derived estimation of LVFP is an independent predictor of MACE, even after adjusting for established risk factors, including LVEF and infarct size. The multivariable model incorporating LVFP_cmr_ exhibited a strong discriminative ability (C-index = 0.767) for predicting MACE, emphasizing the potential utility of this noninvasive hemodynamic parameter in post-AMI risk stratification. Furthermore, the WR analysis revealed that elevated LVFP_cmr_ was associated not only with a higher incidence of adverse events but also with an earlier occurrence of more clinically severe outcomes, providing a severity-weighted validation of its prognostic significance.

In an earlier study, Garg *et al*. [18] proposed and validated the use of CMR-derived pulmonary capillary wedge pressure in patients with AMI, establishing its hemodynamic relevance and associations with adverse remodeling phenotypes derived from routine cine imaging. However, their study focused on cross-sectional associations with ventricular remodeling or filling-pressure surrogates, rather than on long-term clinical outcomes. The present study extends this line of evidence by showing that LVFP_cmr_ independently predicts MACE after primary PCI, even after adjusting for conventional risk factors, LVEF, and infarct size. Hence, this research bridges the gap between physiological plausibility and prognostic validation, confirming that LVFP_cmr_ is a clinically meaningful biomarker for post-AMI risk stratification.

The pathophysiological mechanisms underlying elevated LVFP following AMI provide a plausible explanation for its prognostic significance. Myocardial infarction often results in left-ventricular remodeling, marked by myocardial fibrosis, increased ventricular stiffness, and impaired relaxation, collectively contributing to diastolic dysfunction and elevated filling pressures. As structural correlates of cardiac remodeling, LAV and LVM are directly influenced by hemodynamic stress and are crucial determinants of LVFP [19, 20]. In our cohort, LVFP_cmr_ exhibited a weak yet statistically significant correlation with infarct size (ρ = 0.15, *p* = 0.008). This observation implies that although the extent of the infarct contributes to elevated filling pressures via adverse remodeling, LVFP_cmr_ captures additional physiological dimensions beyond infarct burden alone. Specifically, it probably results from a combined effect of diastolic stiffness, hypertrophy, and atrial remodeling that cannot be entirely explained by myocardial necrosis or scar volume. This observation reinforces the idea that LVFP_cmr_ acts as a combined marker reflecting both structural and functional remodeling after AMI. Elevated LVFP, therefore, functions as a comprehensive marker indicating the severity of ventricular remodeling, the extent of myocardial injury, and the burden of diastolic dysfunction—all of which are important predictors of adverse outcomes [21, 22].

Echocardiography is currently the primary and most extensively utilized noninvasive modality for evaluating left-ventricular function post-AMI. Its advantages include broad availability, bedside applicability, and the capacity to simultaneously provide crucial information on LVEF, valvular disease, and other structural abnormalities [23]. The use of Doppler parameters, especially mitral inflow velocities (E and A waves), mitral annular tissue Doppler velocity (e^′^), left-atrial volume index (LAVi), and tricuspid regurgitation (TR) velocity, integrated within current guideline-recommended multiparameter algorithms, enables a reasonably accurate estimation of LVFP in a large proportion of patients [22, 23]. Particularly, the E/e^′^ ratio has been shown to be a potent predictor of all-cause mortality early after AMI, offering prognostic value independent of, and potentially superior to, LVEF and conventional clinical risk assessment [24]. In contemporary STEMI cohorts with preserved LVEF after primary PCI, a high discharge E/e^′^—and persistently elevated E/e^′^ at 1-year—also identifies patients at increased long-term risk [25]. These findings agree with additional STEMI data demonstrating that elevated E/e^′^ independently predicts long-term adverse outcomes after primary PCI [26]. In addition, a multicenter study by Andersen *et al*. [22] validated the comprehensive echocardiographic approach based on the 2016 guidelines, reporting an accuracy of up to 87% in identifying elevated LVFP compared with invasive measurements, considerably outperforming clinical assessment alone.

Nonetheless, despite significant advancements, echocardiographic estimation of LVFP in patients with AMI continues to face certain limitations. First, image quality and Doppler signal acquisition rely on patient habitus, pulmonary conditions (which may be relevant in the acute phase with pulmonary edema or mechanical ventilation), and operator expertise [23]. Second, the interpretation of specific parameters can be confounded; for instance, E/e^′^ may be unreliable when significant mitral annular calcification, valvular disease, left bundle branch block, or paced rhythm is present. LAVi is less informative in atrial fibrillation [23]. Third, even when adhering to guideline algorithms, LVFP status remains “indeterminate” in a nonnegligible proportion of patients, limiting its decisional value in all individuals [22, 23]. Lastly, reported accuracies can vary between studies and real-world practice, potentially influenced by population selection and operator adherence to protocol [23].

Considering the acknowledged limitations of echocardiography in assessing LVFP, CMR presents clear advantages. It delivers highly reproducible, quantitative assessments of cardiac volumes and mass, which are largely unaffected by acoustic window quality and exhibit lower interoperator variability than Doppler techniques [27]. Recent advances in CMR techniques have extended its use in assessing AMI. Contemporary studies have shown that quantitative CMR markers—such as myocardial strain and four-dimensional (4D) flow—provide prognostic information that surpasses conventional measures, including LVEF and infarct size, highlighting the need for comprehensive functional evaluation in patients with AMI [12, 28]. Compared with these parameters, LVFP_cmr_ provides a uniquely accessible surrogate of diastolic loading conditions derived from routine cine images, enabling integration into standard CMR workflows without additional scan time. Compared with other noninvasive approaches, head-to-head evidence indicates that LVFP_cmr_ can outperform guideline-based TTE algorithms in categorizing elevated filling pressures and can reclassify a substantial proportion of indeterminate or incorrectly classified TTE cases. Importantly, prognostic utility was also noted in follow-up [15]. In addition, in a multimodal heart failure assessment, LVFP_cmr_ showed better diagnostic performance than the echocardiographic E/e^′^ ratio in identifying patients with elevated NT-proBNP, thereby supporting the construct validity of LVFP_cmr_ against an external biochemical standard. Together, these data establish LVFP_cmr_ as a useful and complementary hemodynamic marker that can improve decision-making, especially when Doppler signals are suboptimal or echocardiographic algorithms produce indeterminate results. Head-to-head comparisons have further established higher analyzability and substantially lower interobserver variability for CMR-derived chamber metrics compared with 2D echocardiography [29]. The specific noninvasive LVFP_cmr_ estimation technique used in this research, developed by Garg *et al*. [15], leverages these strengths by utilizing LAV and LVM—parameters robustly measured using CMR—integrated into a linear regression model (LVFP_cmr_ = 6.1352 + (0.07204 × LAV) + (0.02256 × LVM)). By relying on these structural correlates of hemodynamic load (LAV signifying preload/remodeling; LVM indicating afterload/hypertrophy), this method may be less susceptible to the technical difficulties or exclusions affecting specific Doppler signals. Indeed, the original validation asserted the superiority of this model over standard TTE algorithms and its ability to correctly reclassify most TTE-indeterminate cases [15].

**Fig. 7.**
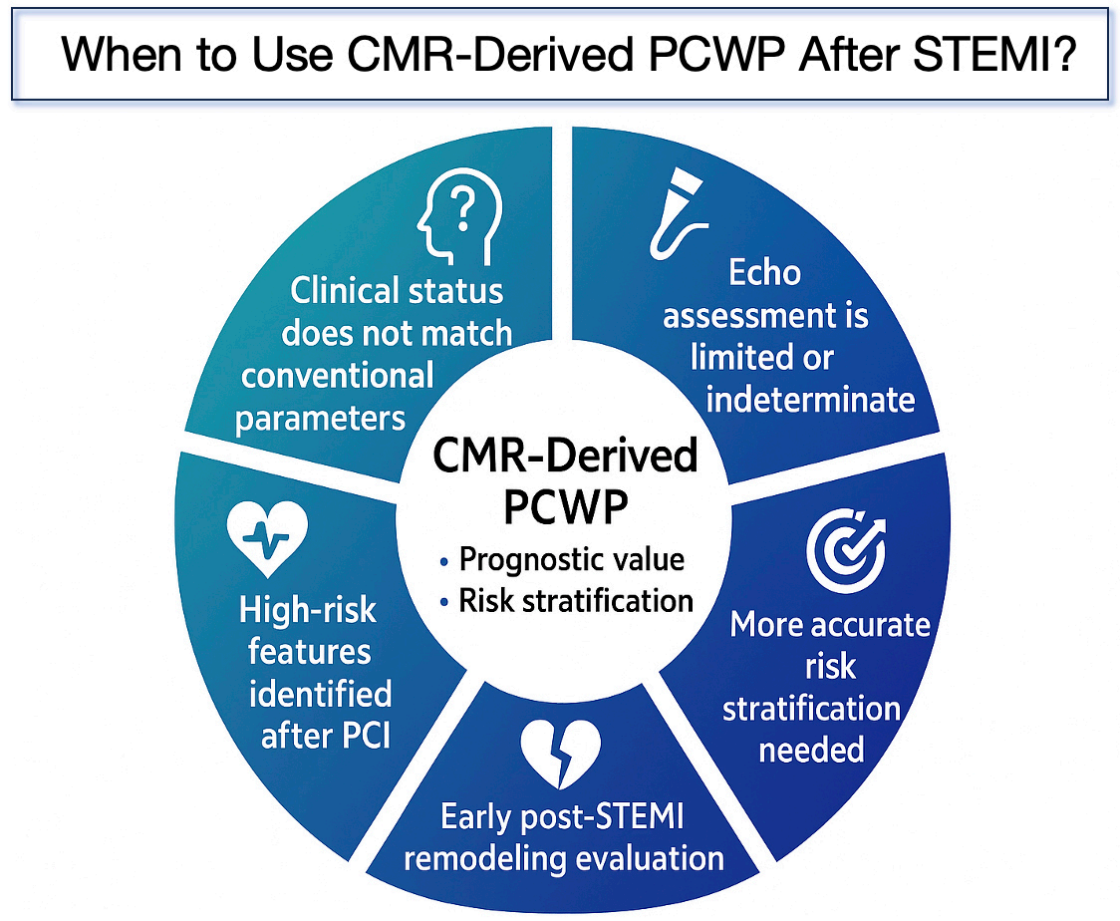
**Clinical scenarios in which CMR-derived PCWP may provide added value after STEMI**. This figure summarizes the major clinical situations where CMR-derived pulmonary capillary wedge pressure (PCWP) may support prognostic evaluation and risk stratification following STEMI, including discordant clinical status, limited or indeterminate echocardiographic assessment, the need for more accurate risk stratification, early post-STEMI remodeling evaluation, and identification of high-risk features after PCI. PCWP, pulmonary capillary wedge pressure; PCI, percutaneous coronary intervention; CMR, cardiovascular magnetic resonance.

However, despite these benefits, estimating LVFP_cmr_ has certain inherent limitations. Although the model demonstrated a reasonable correlation (r = 0.55) with invasive PCWP and achieved good diagnostic accuracy (76%), its R^2^ value of 0.31 shows that LAV and LVM only partly account for the variability in PCWP. Additionally, some proportional bias was observed, which could restrict the accuracy of absolute pressure estimates across the entire range [15]. Furthermore, this specific model does not include advanced CMR metrics (such as tissue characterization or 4D flow) that could improve accuracy, nor can it detect beat-to-beat hemodynamic fluctuations measurable with Doppler. However, given its strengths in reproducibility and integration into a comprehensive assessment, LVFP_cmr_ remains a valuable alternative or complementary method for noninvasive hemodynamic evaluation after AMI, especially when echocardiography is technically difficult or inconclusive. To further test the robustness of the results, a sex-specific sub-analysis was performed using a recently proposed sex-adjusted LVFP_cmr_ equation. The sex-specific model exhibited prognostic behavior very similar to the generic LVFP_cmr_, staying independently linked to MACE and offering comparable discrimination and risk stratification in survival analysis. These findings suggest that while including sex in the equation may enhance physiological accuracy, it does not significantly alter prognostic performance. This observation confirms the reliability of LVFP_cmr_ as a meaningful clinical marker across both sexes in the context of ASTEMI. A visual summary of the key clinical scenarios in which CMR-derived PCWP may provide added value is presented in Fig. 7.

### Limitations

However, our study has certain limitations that should be acknowledged. Our findings, from a retrospective single-center study, are subject to inherent selection and information biases. Patient inclusion was limited to those who underwent clinically indicated CMR after PCI, which might have introduced referral bias toward more stable or evaluable cases. Additionally, the small sample size and limited number of outcome events could have led to model overfitting and decreased statistical robustness. Consequently, the discriminative ability and robustness of our multivariable models should be validated in larger, multicenter prospective studies. Furthermore, this study did not incorporate emerging CMR parameters such as myocardial strain, native T1/T2 mapping, or extracellular volume. Therefore, whether these advanced tissue characterization metrics add incremental prognostic value beyond LVFP_cmr_ could not be assessed, and their mechanistic relevance could not be explored. Future research should incorporate multicenter recruitment with standardized CMR acquisition and postprocessing protocols to minimize institutional bias and enable robust external validation of the prognostic value of LVFP_cmr_.

In this study, a detailed prognostic assessment of post-ASTEMI patients who underwent CMR was conducted, combining conventional clinical parameters with CMR-derived measures, including LVFP_cmr_. The findings established that elevated LVFP_cmr_ was associated with unfavorable outcomes and served as an independent marker for risk stratification. The proposed LVFP_cmr_ metric could be incorporated into post-PCI risk assessment protocols, particularly in patients undergoing early CMR. Its noninvasive estimation allows for routine integration without additional scan time, potentially guiding follow-up intensity and the selection of adjunctive therapy. In clinical practice, an elevated LVFP_cmr_ value may help identify patients who require intensified follow-up, closer monitoring for heart failure progression, or optimization of guideline-directed medical therapy. These findings provide a preliminary framework to identify high-risk post-ASTEMI patients and may inform future studies on surveillance strategies and targeted interventions.

## 5. Conclusions

The findings from this study suggest that LVFP_cmr_, estimated using a model based on LAV and LVM, is a new and independent predictor of MACE in patients recovering from ASTEMI. While these results may aid in clinical risk assessment, they should be interpreted with caution due to the relatively small sample size and the study’s retrospective, single-center design. Larger, prospective, multicenter studies are necessary to validate these findings.

## Availability of Data and Materials

The datasets generated and analyzed during the current study are not publicly available due to patient privacy and institutional regulations but are available from the corresponding author upon reasonable request.
